# In Adults With Severe Psychiatric Disorders, How Does the Option of Assisted Dying Compared with Standard Psychiatric Care or Palliative Care Impact Patient Autonomy, Quality of Life, and Ethical Considerations: A Comprehensive Meta Review

**DOI:** 10.1192/bjo.2025.10122

**Published:** 2025-06-20

**Authors:** Ugo Okafor, Asha Devi Dhandapani, Gaurav Uppal, Khushboo Kansal, Sarah Ahmed

**Affiliations:** ^1^Mater Misericordiae Hospital, Dublin, Ireland; ^2^BCUHB, Wrexham, United Kingdom; ^3^Satyam Hospital, Ludhiana, India; ^4^Nottinghamshire Healthcare NHS, Nottingham, United Kingdom; ^5^KSMC Hospital, Madina, Saudi Arabia

## Abstract

**Aims:** This systematic review sought to compare the effect of assisted dying options on self-determination, patients’ quality of life, and specific/ethical concerns including suicidality for adults with severe psychiatric disorders and psychiatric or palliative care.

**Methods:** The data sources gathered for this review were PubMed, EMBASE, CINAHL and Cochrane databases. The search terms consisted of different forms of assisted dying to which various forms of psychiatric and mental health-related terms were added. The papers were restricted to systematic reviews and meta-analyses as these give high-quality evidence. Out of 343 studies after strict criteria such as ROBINS 1, ROB2 and AMSTAR, only 3 studies qualified for the review. The review centred on adults with severe psychiatric disorders, specifically patients with eating disorders who had assisted dying between 2012 and 2024.

**Results:** The present review estimated that at least 60 individuals with eating disorders who received assisted dying between 2012 and 2024 were reported across 10 peer-reviewed studies and 20 government reports. Clinical rationales for granting assisted dying requests fall into three main domains: non-treatability, prognosis and request of the patient. Most of the reports highlighted two aspects: that the patients had a terminal or untreatable disease, as well as sufficient decision-making abilities. Still, only a few reports were available for the government and many of them failed to provide adequate data on psychiatric conditions.

The review showed that there were significant gaps in reporting assisted deaths for psychiatric patients and ministers questioned accountability and patient safety. Some clinical justifications were void of rigour or evidence indicating the plausibility of the irremediability or lack of decisional capacity in psychiatric relatedness.

**Conclusion:** The findings of this systematic review can be concluded as indicating the lack of procedural clarity and strengthened precaution measures for assisted dying in the field of psychiatry. The results imply the applicability of the ethical principles as well as clinical considerations call for incremental case-by-case analyses. The study should be extended to propose improved reporting systems for assisted dying and to confirm clinical justification for several patients who received help in psychiatric practices, with the consideration of patient rights and safety.

